# Types of Racism and Twitter Users’ Responses Amid the COVID-19 Outbreak: Content Analysis

**DOI:** 10.2196/29183

**Published:** 2022-05-19

**Authors:** Amanda Lloret-Pineda, Yuelu He, Josep Maria Haro, Paula Cristóbal-Narváez

**Affiliations:** 1 Research and Development Unit Parc Sanitari Sant Joan de Déu Sant Boi de Llobregat, Barcelona Spain; 2 Centre for Biomedical Research on Mental Health Madrid Spain

**Keywords:** COVID-19, racism, Chinese, advocacy, Twitter

## Abstract

**Background:**

When the first COVID-19 cases were noticed in China, many racist comments against Chinese individuals spread. As there is a huge need to better comprehend why all of these targeted comments and opinions developed specifically at the start of the outbreak, we sought to carefully examine racism and advocacy efforts on Twitter in the first quarter of 2020 (January 15 to March 3, 2020).

**Objective:**

The first research question aimed to understand the main type of racism displayed on Twitter during the first quarter of 2020. The second research question focused on evaluating Twitter users’ positive and negative responses regarding racism toward Chinese individuals.

**Methods:**

Content analysis of tweets was utilized to address the two research questions. Using the NCapture browser link and NVivo software, tweets in English and Spanish were pulled from the Twitter data stream from January 15 to March 3, 2020. A total of 19,150 tweets were captured using the advanced Twitter search engine with the keywords and hashtags #nosoyunvirus, #imNotAVirus, #ChineseDon’tComeToJapan, #racism, “No soy un virus,” and “Racismo Coronavirus.” After cleaning the data, a total of 402 tweets were codified and analyzed.

**Results:**

The data confirmed clear sentiments of racism against Chinese individuals during the first quarter of 2020. The tweets displayed individual, cultural, and institutional racism. Individual racism was the most commonly reported form of racism, specifically displaying physical and verbal aggression. As a form of resistance, Twitter users created spaces for advocacy and activism. The hashtag “I am not a virus” helped to break stereotypes, prejudice, and discrimination on Twitter.

**Conclusions:**

Advocacy efforts were enormous both inside and outside the Chinese community; an allyship sentiment was fostered by some white users, and an identification with the oppression experienced by the Chinese population was expressed in the Black and Muslim worldwide communities. Activism through social media manifested through art, food sharing, and community support.

## Introduction

### Background

The SARS-CoV-2 (COVID-19) outbreak increasingly generated a sense of xenophobia and hate crimes in Asian communities across the world [1-4]. Some survey-based research has highlighted a positive association between the outbreak of COVID-19 and discrimination experienced among Asians, specifically Chinese individuals [5,6]. In the United States, many racist and xenophobic incidents, notably verbal abuse and physical attacks, have been explicitly on display [5]. Moreover, 1 out of 4 participants in a survey conducted with 1904 Chinese individuals living overseas across 70 countries reported having experienced discrimination amid the pandemic, such as forced layoff, discrimination in rental housing, and abuse in the public arena [7]. In addition, a rising level of a collective sentiment of backlash and racism is manifested in the digital world, such as anti-Chinese hashtags and the use of hateful language [8-11]. In response to racist attacks and stigmatization, Asian groups in France began to use a new hashtag on Twitter, #JeNeSuisPasUnVirus, to engage in telematic activism. As social media provide permanent access to the discussion of topics related to racism, this hashtag promoting activism was rapidly translated into different languages such as English, Spanish, and Chinese (#ImNotAVirus, #NoSoyUnVirus, and #我不是病毒, respectively). The use of these hashtags led to a rapid evolution of discourses and debates on Twitter, unfolding many racial attacks and advocacy efforts toward Chinese individuals. As some existing research suggests, Twitter is a good communication tool to understand public sentiment and behaviors surrounding racial issues triggered by COVID-19 [12-14].

Accordingly, the aim of this study was to investigate the types of racism that developed amid the beginning of the COVID-19 outbreak and how people reacted toward these discourses on Twitter. Analyzing public reactions at the first phase of the pandemic can enable predicting racism and discriminatory actions toward certain ethnic groups in a future pandemic. Based on Tatum’s theory [15], we elaborate an *aims to describe* approach to conceptualize racist online discourses.

### Historical Racism and Oppression Faced by the Chinese

Racial discrimination against Chinese individuals is not a new phenomenon. In the 19th century and the first half of the 20th century, the Chinese became underpaid small business owners in the Americas. In the host society, they were segregated in Chinatowns and experienced constant harassment from white Americans [16]. As a result, the term “yellow peril” was used to describe Chinese migrants as a threat to the white civilization [17]. Australia also prohibited Chinese migrant laborers from entering the country and denied them a civic status, as they were seen as fierce competitors to local economies. These events were rooted again in white supremacist cultural ideas [18]. A relevant date in history was 1882, when the Chinese Exclusion Act was approved in the United States. This new order explicitly denied access to all Chinese laborers from the country considering their race and ethnicity. Due to China’s restriction of labor migration, the US government recruited cheap laborers from other Asian countries such as Japan, India, Korea, and the Philippines; however, this resulted in new waves of racial exclusion [19]. In the second half of the 20th century, with the arrival of migrants from the global South, there has been a rise of xenophobia toward Chinese migrants in Europe. In Great Britain, Chinese individuals were considered as “Red threats,” “Blue ants,” and “laundry-lords” [17]. In Italy, the Chinese have been stereotyped as “making too much noise” and “stealing jobs from locals,” which has provoked a hostile sentiment toward the Chinese diaspora [20]. After the severe acute respiratory syndrome (SARS) outbreak in 2003, the Chinese experienced discrimination in many countries such as Canada, the United States, and the United Kingdom [21,22]. With the outbreak of COVID-19, these racial dynamics might increase. A recent study analyzed the role of stigmatizing words on Twitter during the first quarter of 2020 [9], showing that the novel respiratory virus was predominantly called the “Chinese virus.” Other nomenclatures such as “Wuhan coronavirus” or “China coronavirus” were constantly used, which acted as an accelerator for stigmatization, bias, and backlash.

### Tatum’s Antiracist Theorization

In the present study, Twitter messages (tweets) were classified into different typologies of racism to understand how systems interplay during the outbreak of COVID-19–related anti-Chinese attacks. In this regard, alongside the historical context displayed, Tatum’s [15] theorization is a social justice–oriented framework that strengthens social and political leaders’ capacity to put in motion antiracist social policies and practices considering all possible levels of oppression. With the collection of a variety of tweets, it is possible to gain a better understanding of the social implications of racism and the antiracist practices to anticipate. The main important themes found in the study were individual racism (divided by active racism and inactive racism forms), cultural racism, and institutional racism. *Individual racism* has been defined as all beliefs of prejudice, power balances, attitudes, and actions that support and perpetuate racism between individuals [15]. Cultural racism is based on cultural images and messages that affirm whites’ assumed superiority. By contrast, institutional racism represents the network of institutional structures, policies, and practices that create advantages and benefits for whites, while maintaining discrimination, oppression, and disadvantage for people from targeted racial groups [23].

Bell [24] explains that some individuals overtly use racism. In acts of overt racism, the oppressor is aware of the use of prejudice and discrimination. Racism can occur on an unconscious level and can be both active and passive [23]. Tatum [15] uses the terms “active racism” and “inactive racism” to talk about this level of consciousness, adapting the concept to having an explicit or an implicit goal to racism. In particular, active racism comprises all of the intentional actions with a stated or explicit goal to maintain a system of racism and the oppression of those in targeted racial groups [15]. One of the main beliefs for perpetuating active racism is that white individuals are superior to other ethnic groups in terms of culture and values [23]. Inactive racism is defined as beliefs, attitudes, and actions that actively contribute to the maintenance of racism without openly using violence or oppression [15]. In other words, inactive racism is maintained through attitudes, beliefs, and behaviors that support the system of racism, racial prejudice, and racial dominance [23]. Inactive racism has also been termed “passive racism.” Tatum [15] conveyed that some clear examples of this phenomenon are seen when a racist joke is told and receptors laugh, when conversations on difficult race-related issues are silenced, or when Black candidates are eliminated in hiring processes.

With respect to cultural racism, Tatum [15] supports the idea that we cannot always identify who is responsible for an individual act, as society is behind the maintenance of certain prejudices and stigmas. Other authors such as Wijesinghe et al [23] added that the conceptualization of cultural racism needs to include the notion of inferiority or devaluation. They further argued that institutional racism is often confused by “rights” and the system of advantages created for white individuals [23].

### Social Media Activism and Advocacy

*Hashtag activism* is a new term that was coined to describe protests on social media. Users can report their personal history or make social and political claims by tweeting, retweeting, or commenting on others’ tweets [25]. For example, the #BlackLivesMatter movement triggered by the death of George Zimmerman in 2012 rapidly evolved into demonstrations in the virtual space. Similarly, during the initial phase of the COVID-19 pandemic, various racial attacks toward the Chinese were reported around the globe. Consistently, Twitter users utilized the hashtags #JeNeSuisPasUnVirus and #I’mNotAVirus as an attempt to unfold the existing racist practices. To the best of our knowledge, this is the first study to directly focus on the racist discourse toward the Chinese ethnicity, along with actual advocacy and activism actions displayed on Twitter during the COVID-19 outbreak.

The aims of this study were to describe (1) which type of racism was displayed against Chinese individuals amid the COVID-19 outbreak (first quarter of 2020), and (2) how Twitter users reacted to news, posts, and tweets that had a positive or negative sentiment toward Chinese individuals.

As a hypothesis, it was expected that due to the worldwide situation of discrimination and hate against the Chinese during the new coronavirus outbreak in Wuhan, tweets in the first quarter of 2020 would exhibit verbal and physical aggression forms of racism against Chinese individuals (ie, individual racism with an emphasis on active racism). We further hypothesized that even with the racist dialog exposed on Twitter, users would paradoxically create advocacy and activism spaces on this social media platform.

## Methods

### Design

Content analysis of tweets was utilized to address our aims. Content analyses transform the symbolic content of a document, such as words and images, into a systematic set of categories and codes [26].

### Data

#### Collection of Tweets

Using the NCapture browser link and NVivo software, tweets in English and Spanish were pulled from the Twitter data stream from January 15 to March 3, 2020. A tweet is a limited virtual message of 280 words [26]. Twitter is a microblogging site in which users engage in real-time messages and can connect with other users by following the feed of other user accounts [26]. A total of 19,150 tweets were captured using the advanced Twitter search engine with the keywords and hashtags #nosoyunvirus, #imNotAVirus, #ChineseDon’tComeToJapan, #racism, “No soy un virus,” and “Racismo Coronavirus.” From the full Excel data set, 1173 tweets were repeated and consequently excluded. A total of 17,575 tweets were not related to our research questions’ topics. After reading all of the tweets, an exclusion criteria checklist was created to tag the tweets (1) not considering the coronavirus outbreak; (2) not related to Chinese and Asian discrimination; and (3) targeting other topics such as immigration in the United States, worldwide politics, new scientific facts related COVID-19, and general opinions about the pandemic and the disease. Finally, 402 tweets were included and coded by three researchers.

#### Themes

The main subcodes for *active racism* include *physical aggression*, *verbal aggression*, *change in relationships*, *rejection*, and *bullying*. Relevant codes for *inactive racism* were *prejudices*, *rumors*, and *jokes*. Separately, a new category termed “*internalized domination*” emerged.

For the second aim, gauging Twitter users’ reactions to the anti-Chinese sentiment, the main topics of discussion were *advocacy* and *activism*. Different subcodes for advocacy emerged, including *Black and Muslim identification with Chinese discrimination*; *white allyship*; and *the break of stereotypes, prejudice, and discrimination* as a form of advocacy using Twitter. In an activism effort, subcodes such as an *antiracist fight* were revealed. We also detected some tweets related to unintentionally internalized racist discourses by certain Chinese individuals.

### Ethical Considerations

No application for an ethical review board assessment was submitted as the study only involved preanalysis of secondary data with no identifying information.

### Coding of Tweets

For the validity of the data process, a codebook based on axial coding was developed, along with instructions on how to use the code and the associated definitions. Before beginning the process, two research team meetings were necessary to determine and clarify the meaning of the codes and the coding strategy. The idea was to be immersed in an intense analysis surrounding a few specific sets of codes and categories, and to preserve selectiveness [27]. The research team reviewed the tweets using an inductive approach to finalize the codebook. As theories were identified behind the tweets, definitions included relevant racism conceptual frameworks, specifically based on Tatum’s [15] concepts. Memos were also used in the analysis processes to improve the gathering of information. Table 1 provides the relevant codes and their definitions.

Once all of the tweets were coded, the codes were compared between two researchers to discuss possible conflicts using a Google Sheet. In the case of disagreement, each remaining tweet was coded independently again by at least two members of the team. Differences were reconciled through discussion between the two coders until reaching 100% agreement. The rate of code conflict was 38/402 (9.4%) tweets for research question 1 and 23/402 (5.7%) tweets for research question 2. To estimate the intercoder reliability, we selected a strategy of group consent rather than statistical consent. Group consent was based on establishing an agreement between coders during the decision-making process and in-depth discussions of the tweets’ content.

**Table 1:** Relevant codes, subcodes, and definitions.

Codes and subcodes			Definition
**Active racism**			
	Physical aggression	Behavior causing or threatening physical harm toward others, including hitting, kicking, biting, using weapons, and breaking toys or other possessions	
	Verbal aggression	Message that attacks self-concept, including insults or name-calling, shouting, teasing, and mockery (eg, “yellow peril” or “Alerte Jaune”)	
	Change in relationships	Change in relationships in a negative/derogatory manner; always takes into consideration that the prior established relationship was based on respect	
	Rejection	Refusing someone for being Chinese without a previous relationship	
	Bullying	Harm, intimidation, or coercion of an individual in the school context	
**Inactive racism**			
	Prejudice	Preconceived judgment or opinion, usually based on limited information	
	Rumors	Actual circulating story of uncertain or doubtful truth	
	Jokes	Use of humor to maintain racism	
Cultural racism			Cultural images and messages that assumed superiority of whites and the assumed inferiority of Chinese individuals, along with negative stereotypes as oversimplified generalizations about the Chinese
Institutional racism			The network of institutional structures, policies, and practices that create advantages and benefits for whites and disadvantage for targeted racial groups, including the Chinese
Internalized domination			Members of the agent group (whites) accept their socially superior status as normal and deserved
Advocacy			Twitter users consciously defending an idea or a purpose; being involved in an activity or action with the idea of influencing social change
White ally			White person who actively works to eliminate racism in social media
**Advocacy**			
	Asian food sharing	Food sharing in the community to defend the cause that Chinese individuals are not viruses	
	Community support	Supporting through hug performances and donations	
**Engage in racist discourse**			
	Intentionally racist	When the message is communicated with the explicit goal to hurt or harm another person	
	Unintentionally racist	Online messages where the communicator is not aware that their arguments have a racist impact	
	Strategically racist	Using discourse to make a point that is part of a broader argument designed to persuade the public or gain political support but is still racist	

## Results

### Research Question 1: Racism Toward the Chinese Amid the COVID-19 Outbreak

#### Overview

A total of 402 tweets displayed some racist content. To address our first aim, we considered whether the racism occurred at the individual, cultural, or institutional level. With Tatum’s [15] conceptualization in mind, the vast majority of tweets showed individual racism (100 tweets) and cultural racism (41 tweets), whereas institutional racism tweets were limited (9 tweets). Table 2 summarizes the codes obtained, which are described in further detail below.

**Table 2:** Summary of the codes for tweets related to racism against the Chinese at the beginning of the COVID-19 outbreak.

Codes and subcodes					Example tweet
**Individual racism (n=100)**					
	**Active racism (n=68)**				
		Physical aggression (n=39)	Also seeing some news reports that people who are definitely “Not Racist” around the world are attacking Asian people who are not from China, because #COVID19 started in China. #Racism		
		Verbal aggression (n=28)	Yesterday when I was in this small town, several people referred to us “Coronavirus”. When they realized we understood Spanish, they stopped chatting... Then, they rationalized their actions by claiming that they didn’t say anything about.		
		Change in relationships (n=3)	Due to perpetuation of #racism like this, my 13 yr old + teammate endured the Judge of their #debate round avoid her assigned seat next to them for a seat across the room from them.		
		Rejection (n=23)	I did a test on the bus the other day, I was sneezing all the time on the trip, no one told me off, but as soon as Asians are associated with any symptoms people turn away #auspol #AuspolSoCorrupt.		
		Bullying (n=5)	This is Constantine, a Chinese student studying in Oz. He was recently beaten up and verbally abused with #racism all cuz he spoke #Chinese. The racial hysteria as a result of the #CoronavirusOutbreak has made this country unsafe for students.		
	**Inactive racism (n=32)**				
		Prejudice (n=15)	Let’s be clear. #COVID2019 or #coronavirus is not the common cold. It is not carried by solely asians. It is a huge threat but this does not mean we should stop supporting Asian businesses. I will be having Chinese food for lunch. #racism #fearmongering		
		Rumors (n=14)	Just lost a few brain cells listening to that ‘instagram influencer’ on your Thurs show.Also, is it not reasonable to assume that those working in /patronizing Chinese restaurants have increased chance of traveling or being in contact with persons traveling to China? #racism?		
		Jokes (n=5)	What I enjoy best is the concept: I catch the virus and then become entirely immune, so I can travel about Wuhan liking Chinese and everything will be OK!		
**Cultural racism (n=41)**					
	Journalist photos (n=12)			The @nypost once again demonstrated their poor journalistic standards by placing a picture of asians in Flushing with their article regarding the first confirmed case of the corona virus. #coronavirus #Coronavirusnyc #WorldHealthOrganization #nypost #racism	
	Yellow peril (n=2)			None of that shields me or anyone else. I’ m still just a target because I’ m east Asian. #JeNeSuisPasUnVirus #ImNotAVirus #Coronavirus #COVID19 ‘They yelled Coronavirus’ – East Asian attack victim speaks of fear	
Institutional racism (n=9)					What’s scary is that just because Mexico has 4 confirmed cases of Coronavirus, Trump wants to close down the border, meanwhile the US has 74 confirmed cases of Coronavirus, so if anything Mexico should close down their border. #coronavirus #TrumpVirus #racism #COVID19US
Internalized domination (n=6)					I’m just saying. If this virus started in Europe or America from some Caucasian folks, y’all wouldn’t say a thing to attack them physically or verbally #coronavirus #COVID2019 #racism

#### Individual Racism

##### Active Racism

Instances of individual racism were broken down into active racism and inactive racism (Table 2). A total of 68 tweets exhibited active racism, characterized by the main subcodes physical aggression, verbal aggression, change in relationships, rejection, and bullying.

Manifestations of physical aggression were diverse and included attacking, assaulting, and beating: “He is an Italian-Chinese man who was violently assaulted for racial reasons.” Some tweets exhibited a combination of physical aggression and verbal aggression in their content, underlying how physical and verbal aggressions go hand-in-hand (n=14):

A student in Great Britain was physically attacked by a group of people yelling him “#coronavirus” and “I don’t want #coronavirus here”. This is the outcome. This #racism is no longer a joke

Verbal aggression was frequent, especially name-calling. The most commonly used insults were “Corona” and “Coronavirus,” although allegations such as “Yellow peril,” “Yellow Alert,” and “Yellow Face” also manifested:

Yesterday when I was in this small town, several people referred to us “Coronavirus”. When they realized we understood Spanish, they stopped chatting... Then, they rationalized their actions by claiming that they didn’t say anything about

Change in relationships as part of active racism was also evident: “An Asian-Australian woman whose family has been in this city for a long time: I had a friend who rejected to have a drink with me.” Verbal aggression was also associated with this behavior:

As much as I admire this guy, this was a mistake. I’m sure he’s regretting it, but let’s deal with it, he’s not the only one. People are refusing to get their meals at Chinese restaurants, Chinese children are being segregated at their educational centers, and so on....

This type of rejection was also frequent in the medical field, including rejecting doctors that appear to be Asian: “Patients at an Australian hospital are rejecting to be treated by Asian doctors, because they are afraid of getting #COVID19.” Chinese restaurants also lost business:

On my way home tonight, all take-aways were crowded except the three Chinese restaurants. “Next people will quit eating pasta!” I told to my daughter. Ok, be cautious. This is just obvious racism.

Manifestations of active racism in the school context were also present. Signs of bullying such as intimidation, coercion, and harmful behaviors were detected:

A Chinese student living in Australia was recently attacked and verbally abused with racism because he spoke Chinese. The Coronavirus outbreak has generated racial panic, making our country dangerous for students. Please, donate.

Bullying was also associated with rejection in certain cases such as Chinese children being segregated in educational centers (Table 2).

##### Inactive Racism

Inactive racism denoted the use of *prejudice*, the *promotion of “rumors,”* and racism through disrespectful *“jokes”* (Table 2).

The concept of “I am not a virus” emerged from the idea that wearing a mask is having the virus. On Twitter, the mathematical form “ASIAN+MASK=VIRUS” is recurrent to explain prejudice. The hashtag “I am not a virus” appeared to break this prejudice or as a form of advocacy:

I got these from a New Zeeland webpage section. It is racist to refuse to breathe the same air as Asian people when making an assumption that Chinese are infected based off a five second glimpse.

During the first quarter of 2020, rumors about Chinese individuals started to appear. Several untruthful stories were circulating, such as that all Chinese individuals have the virus and that they are dangerous:

A twitter user assumes only that Coronavirus primary affects Asians, so he is unconcerned. The racism and cruelty are always a thing. Moreover, his president informed him it will be gone by April so he is just counting down days until it’s done.

More specifically, rumors affected Chinese individuals who work in Chinese restaurants, as there was a rumor about Chinese employees getting coronavirus: “It is not congruent to assume that those working in Chinese restaurants have increased chance of traveling or being in contact with people traveling to China.”

A Twitter user confronted this rumor:

Listening to an ‘Instagram influencer’ on a Thursday show, I just lost a few brain cells. It is not normal to presume that folks who work in Chinese restaurants have a higher possibility of traveling to China or coming into contact with people who are traveling there.

Rumors about the Chinese eating pets were also frequent. One Twitter member attempted to educate users on this rumor:

It’s time to address the notion that people in China eat their pets. It has been stated to me since I was a child, but this is not true; this practice does not exist in China, and only occurs in really poor areas. Chinese are humans, and they also have pets.

Some individuals used harmful jokes to attack Chinese individuals. An example is provided: “What I enjoy best is the concept: I catch the virus and then become entirely immune, so I can travel about Wuhan liking Chinese and everything will be OK!”

#### Cultural Racism

Cultural racism was exhibited in general messages, but also through the subcodes *journalism photos* and *promoting “Yellow Peril” stereotypes* (Table 2): “It appears that other ethnicities enjoy bat soup as well. Please don’t make a racial attack on them right now. This Twitter user prefers them to be fresh.”

The journalism photos code was used when detecting photos and images in the public media that contained discriminative or prejudiced content toward Chinese individuals. For instance, some Twitter users denounced: “Why every Western media always utilizes images of Asians when they announce that coronavirus has been detected in Europe?” In this regard, the *journalism photos* code complemented the *advocacy* code: “The @nypost once again proved their terrible journalistic standards by accompanying an article on the first confirmed case of the corona virus with a photo of Asians in New York State.”

Specifically, subcodes on how journalism photos reflect a cultural racist image (ie, Chinese people wear masks because they are infected) can be appreciated regularly during this period. Another important subcode was *yellow peril*, as this historical idea strongly persisted during the first quarter of 2020 on different cultural racist tweets:

Thank you to these specific Twitter users for inviting me to speak on this fantastic podcast regarding the coronavirus outbreak and racism. The COVID-19 racism is just one manifestation of Australia’s long-standing yellow peril and anti-Chinese sentiment

#### Institutional Racism

Institutional racism was less frequently mentioned, with a total of 9 tweets pointing to this problem. The institutional racism issue was generally brought up indirectly: “Universities have already lost 100,000 Chinese students; if Indian parents learn of this, they may withdraw the remaining 100,000 students.”

#### Internalized Domination

Six tweets demonstrated the existence of internalized domination. The following comment reflects how a white US Twitter user indirectly treats Chinese and Iranian individuals as inferior:

It’s funny how the death rate from the coronavirus is higher in heavily inhabited POC [people of color] areas like Iran and China. However, in nations where Whites are the majority, such as the United States and Italy, the rate is quite low.

Another tweet was found in which a user expressed a sentiment of superiority of Black individuals to Asians:

I witnessed a black man abusing an elderly Chinese man. Many Black folks have historically waved the flag in support of anti-racist causes. This time, though, Black people are against Asians.

However, internalized domination was also criticized by another Twitter user: “How come you don’t think about how much your predecessors suffered because of prejudice when you do the same to other races?”

### Research Question 2: Advocacy and Activism Through Social Media

#### Advocacy

After confirming that racism was displayed on Twitter at different levels, we next inquired about the most common user responses to this racist sentiment on social media, as highlighted by the high number of tweets related to *advocacy* and *activism* (Table 3).

**Table 3:** Summary of tweets associated with advocacy and activism against Chinese racist sentiment.

Code and subcode		Example
**Advocacy (n=298)**		
	Break stereotypes, prejudice, and discrimination (n=100)	I don’t wanna be ‘that guy’ but..This is what I mean about the double standards of #racism. If a white person had written that about a black person then #Twitter would be in meltdown. Is #coronavirus #racist? Are #scientists racist?No that Tweet is. #Equality is EQUAL.
	White allies (n=33)	Please stop the #racism, #Xenophobia, and open hatred against Asians because of the #coronavirus. A virus is not and should not be a means to discriminate and Other human beings.
	Black identification (n=12)	Some folks whose minds are so debased have started exhibiting stereotypical disposition towards Chinese whom at this critical time need the world’s support to overcome #coronavirus #CoronaVirusUpdates #COVID–19 #racism #ourHealthMatters #stopracism #Health
	Muslim identification (n=9)	#FreedomOfSpeech vs #Racism. #Sinophobia is the new #Islamaphobia or #AntiSeminitism
Activism (n=43)		#Coronavirus: #Wuhan natives in US unite to support their city during crisis New York people order fund raising for #medical supplies; But some NY Chinese encounter virus sparked #racism

Some subcodes help to understand who was involved in these advocacy efforts. The code “toward others” (149 tweets) helped to determine that not only Chinese individuals were involved in this process, but that non-Chinese individuals also advocated for racism experienced by the Chinese amid the coronavirus outbreak. Chinese users also advocated for themselves (64 tweets). In some cases, advocacy efforts were not only about oneself on an individual level (45 tweets), as some Chinese individuals included their Chinese community or Asian community as a whole in their advocacy tweets. The following tweet reflects the awareness of advocating against racism toward Chinese individuals:

The only actual threat of the corona virus is the prejudice directed at any Asian person, which many people now believe is acceptable. While in the train, a woman films a racist coronavirus rant.

Most of the tweets with an advocacy goal were meant to break stereotypes, prejudice, and discrimination (Table 3):

In the wake of the #coronavirus outbreak, the Human Rights Commission has offered advice: wash your hands and avoid being #racist and #xenophobic. Coronavirus makes no distinction between your skin color, whether you are black or white. We need to pray for each other. #racism #blacklivesmatter

White allies played an active social media role to deconstruct ideas about the Chinese as viruses (33 tweets). As a team, we faced some difficulties in identifying a Twitter user’s nationality and/or ethnicity; if this information was not explicitly stated on their Twitter profile, we did not make any inferences. The same applied to the codes *Muslim identification* and *Black identification*:

People on my Facebook page believe that China produced the Corona virus as a type of biological warfare. They cite SARS and avian flu as examples. Why would they do anything like that to their own economy? I'm starting to lose faith in human intelligence.

The Black identification code emerged as some Black Twitter members—who claimed their chosen identity in their Twitter profile—identified with the struggle of racism experienced by the Chinese amid the coronavirus outbreak. A total of 12 tweets were coded as Black identification: “Some people with degraded minds have started acting stereotypically against Chinese people who, at this vital time, they need the world’s support.”

A total of 12 tweets were coded as Black identification. An example is given: “Some people with degraded minds have started acting stereotypically against Chinese people who, at this vital time, they need the world’s support.”

A hypothesis that may need to be explored in future studies is the possible relationship between the discrimination faced by Black communities during the AIDS and Ebola outbreaks as a parallel to Chinese discrimination during the COVID-19 pandemic. Unfortunately, our study’s qualitative data were too vague to establish any solid relationship on why Black individuals felt particularly empathetic with respect to Chinese racist attacks. Notably, in some countries, including the United States, a recent allyship has been exhibited with the hashtags #asians4blacks during the recent #blacklivesmatter movements in 2020. This code is thus a processor for the reciprocal allyship recently reported.

Muslims also appear to identify with the struggle of racism experienced by the Chinese amid the coronavirus outbreak (9 tweets). When a member of another group experienced oppression, empathy emerged with other communities: “I am mixed race (Arab) and Arabs are racist, they treat Black, desi, east & southeast Asians unrespectfully.”

#### Activism

A total of 298 tweets were coded as showing activism. Different types of actions were coded under *Antiracist fight* (n=43), including *art* creation, *Asian food sharing*, and *community support* through hug performances and donations (Table 3). In the following example, a Chinese student is asking for donations via GoFundMe. He was assaulted, and is asking for reparations denouncing his situation:

*A Chinese student living in Australia was recently attacked and verbally abused with racism because he spoke Chinese. The Coronavirus outbreak has generated racial panic, making our country dangerous for students. Please, donate.*

The following initiative used art to combat racism:

Online exhibitions, live museum visits, and special social media events are the answers that Iranian museums promoted to avoid COVID-19. We have initiated a movements against anti-Asian racism within the museums.

In Toronto, a food initiative to fight racism emerged:

This is also true here in Toronto. I’ve recently made it a point to eat out and order takeaway from an Asian restaurant whenever possible. People will arrive at their favorite late-night eateries in the summer and be perplexed as to why they are closed.

Similarly, in the United States, a Twitter user was promoting eating Chinese food for lunch as the rejection against Chinese businesses increased:

Let’s be clear about something. The coronavirus, also known as COVID-19, is not the same as the common cold. Not only Asians get them. It’s a significant threat, but it doesn’t mean we shouldn’t continue to support Asian businesses. For lunch, I’m going to get Chinese food. #fearmongering #racism.

### Racist Discourse on Twitter

Apart from the advocacy tweets and activist actions again racism, there were also some tweets demonstrating racist discourse (n=13). Two tweets were intentionally racist and 11 tweets were classified as *unintentionally raci*st. None of the tweets analyzed was codified as strategically racist. The following tweet exemplifies an intentionally racist discourse, as the message is communicated with the explicit goal to hurt, harm, or discredit the Chinese community [28]: “Prepare for a Coronavirus pandemic around the World. Thank you, China. Up yours to everyone who claimed racism. Your hands will be stained with blood!”

By contrast, the following Twitter member engaged in unintentionally racist discourse, as they were not aware or concerned that their arguments could have a racist impact [28]:

People who believe that avoiding vulnerable or compromised communities is #racist deserve no respect. Why do you think prejudice evolved in humans? To keep themselves safe from something far worse! Everyone: To avoid a plague, avoid sick individuals like the plague!

## Discussion

### Principal Findings

The present work investigated the typology of racism manifested against Chinese individuals amid the COVID-19 outbreak in the first quarter of 2020, and how Twitter users reacted toward racism against Chinese individuals on this specific social network. As main findings, the data support the presence of individual, cultural, and institutional racism against Chinese individuals. Individual racism was the most reported form of racism, especially conveying physical and verbal aggression. Positive reactions that supported the Chinese community were more widely shared. As a form of resistance against racism, Twitter users reacted by engaging in advocacy and activism within their social network. Advocacy was claimed with the hashtag “I am not a virus,” which served to break stereotypes, prejudice, and discrimination. Activism messages showed resistance through art, food sharing, and community support.

Notably, the interpretation of these findings are relevant at three levels: (1) Twitter messages can predict acts of racism and discrimination in advance; (2) Twitter posts help to detect different forms of racism, which works as a tool to fight racism per se; and (3) the online advocacy and allyship work conducted on Twitter can help to break stereotypes in the virtual environment and real world simultaneously. For this reason, the descriptive data provided in this study can strengthen social leaders’ capacity to predict racism in the public arena, and begin to implement policies and practices before acts of racism occur, taking into consideration a systematic level for each of the antiracist interventions.

Despite the limited literature on this topic, our study findings line up with the most recent work available [5,7,8,29]. Verbal abuse and physical aggression were the most common forms of racism identified during the outbreak of COVID-19. He et al [7] also found a range of discriminatory acts that varied from verbal abuse to violent attacks. Similarly, we found that discrimination against Chinese individuals occurred in different contexts, with narratives displaying racism on public transportation and schools being prominent. Criss et al [8] reported that racist statements covered overt and subtle expressions against Black, Latinx, and Chinese individuals. However, they did not analyze the direct impact of the COVID-19 outbreak targeting only Chinese individuals [8]. Nevertheless this study and previous work showed that subtle, or inactive, forms of racism manifest in our communities.

Data from 2020 also suggest that Chinese individuals living abroad experienced stigmatization during the COVID-19 pandemic, ranging from overt racism to covert microaggressions [29]. We identified expressions of racism both in overt and subtle forms, including from international students, endorsing previous data regarding this topic. For example, as an overt manner of manifesting racism, Ma and Zhan [29] noticed that Chinese individuals reported verbal abuse, hearing “coronavirus” yelled at them. In our study, Chinese individuals also reported the same type of overt verbal aggression. It is also relevant to highlight that Criss et al [8] detected a vast number of stereotypes against Chinese individuals. Even though their study was not strictly targeted to understanding anti-Chinese racism during the COVID-19 outbreak, they found that the Chinese were highly stigmatized based on their physical features [29]. In our study, these manifestations on Twitter were also noted in the context of name-calling.

Abd-Alrazaq et al [30] analyzed tweets to identify the top concerns of Twitter users. Although the main topic of the study was not racism per se, this subject emerged indirectly as they found a clear increase of racism against Chinese individuals as a form of distress. Their data seem to align with the idea that the Chinese have been impacted by racism during the COVID-19 outbreak. Specifically, the current study demonstrates that racism against Chinese individuals during the COVID-19 outbreak was displayed in many manifestations, including physical, verbal aggressions, and bullying, as reported in past studies, and well-established relationships also changed. Rejection or refusing someone simply for being Chinese or Asian was also common. Rumors and jokes were actively present as a form of uncovered racism. Thus, this study denotes that racism occurs on a broad spectrum and at different system levels—individual, cultural, and institutional—and all forms must be considered.

One of this study’s main strengths is the use of Twitter as a social media network to analyze manifestations of racism against the Chinese amid the COVID-19 outbreak. Twitter was selected as it constitutes a developing setting in which racism and related stress manifest [9]. Twitter is also an important social media platform because it includes information for users to express their worries, opinions, and feeling about the pandemic [8]. Additionally, in recent years, most online social media movements and social forms of denouncement originated or were consolidated on Twitter (eg, #Blacklivesmatter, #sayhername, #asians4blacks).

Nevertheless, limitations should be considered when interpreting the results of this social media study. Although we aimed to analyze tweets in different languages, we were only able to focus on tweets in English and Spanish. As the process of intercoder reliability requires two or more researchers speaking the same language, we could not include tweets in Japanese, Chinese, German, Italian, or French. To prevent bias, we excluded the analysis of certain hashtags such as #*ichbinkeinvirus*, #*jenesuispasunvirus*, and #*IoNonSonoUnVirus*. Furthermore, a small number of tweets were not available (as some users erased a tweet or Twitter blocked specific messages). In this sense, some tweets no longer existed once captured by NCapture and put in the Excel sheet.

Another major point is that most of the studies on this topic tend to be general when using terminology related to racism. For example, the main goal of the study of Criss et al [8] was to evaluate how information about the virus originated and how fake news spread and fluctuated on Twitter along with its impacts. In their work, racism was a subjacent topic. In this sense, it was quite complex to find research that gathered information directly on racism, specifically against the Chinese, on Twitter during the COVID-19 outbreak. Similarly, the target population of related studies was also broader, and the methodology used varies from qualitative to quantitative designs. As the online hashtag “I am not a virus” was still new when we began this research, and we only analyzed tweets from January 15 until March 3, 2020, we cannot evaluate the connection with subsequent events.

Finally, it is important to mention that when some of the first COVID-19 cases were identified in Italy, discrimination against the Chinese had already been reported on Twitter. As the oppression increased against Chinese individuals, three tweets exhibited how some individuals developed an anti-Italian sentiment and discriminated against Italians during the novel coronavirus outbreak. However, we did not find instances of anti-Italian sentiment systematically in this research. Nevertheless, it is interesting to consider these types of tweets to understand how a group’s oppression can evolve to the oppression of another group during a pandemic. Future studies should take a closer look at this issue and how the relationship between communities evolves. Additionally, we noticed that the terminology used to define the different classifications of racism differs among authors. We used Tatum’s [15] conceptual framework to guide this study; however, no theoretical perspectives were explicitly identified in the other related articles reviewed. Given this situation, it would be convenient to begin theoretically based conversations between researchers to establish a common ground and standardized terminology for future studies.

### Conclusions

Most of the tweets analyzed exhibited individual racism from January 15 to March 3, 2020. Specifically, physical and verbal aggressions were denounced during this period, with active forms of racism being more widespread. At lower intensity, rejection as an active form of racism was highlighted (eg, canceling taxi reservations only once seeing a person’s Chinese name or rejecting doctors that appear to be Asian in the medical field). The hashtag “I am not a virus” was used during the first quarter of 2020 as a form of antiracist advocacy. Twitter is used as a form of advocacy to denounce mainly physical and verbal aggressions. The hashtag “I am not a virus” helped break stereotypes, prejudice, and Twitter discrimination. Allyship relationships were also evident, including white, Black, and Muslim allyship. Activism through social media manifested through art, food sharing, and community support.

As social implications and considerations for the future, policies and practices must be concentrated on preventing physical aggression and verbal abuse against the Chinese. This is necessary to fight racism considering the different systems implied, as antiracist interventions have to be targeted at the individual, cultural, and institutional levels. Covert manifestations are also present in society, and thus interventions in this direction must not be forgotten. Overall, this study highlights that Twitter could be a useful tool for advocacy and activism, helping to break stereotypes, prejudice, and discrimination at a population level. Thus, social interventions should consider both real life and cyberspace for antiracism education in the general population.

## Abbreviations

SARS: severe acute respiratory syndrome
